# A survey of smoking prevalence and interest in quitting among social and community service organisation clients in Australia: a unique opportunity for reaching the disadvantaged

**DOI:** 10.1186/1471-2458-11-827

**Published:** 2011-10-26

**Authors:** Jamie Bryant, Billie Bonevski, Christine Paul

**Affiliations:** 1 Priority Research Centre for Health Behaviour, School of Medicine & Public Health, University of Newcastle, Hunter Medical Research Institute. Room 230A, Level 2, David Maddison Building, Callaghan, NSW, 2308, Australia; 2 Health Behaviour Research Group, Priority Research Centre for Health Behaviour, School of Medicine & Public Health, University of Newcastle, Hunter Medical Research Institute. Room 268 Level 2, David Maddison Building, Callaghan, NSW, 2308, Australia

## Abstract

**Background:**

Social and community service organisations (SCSOs) are non-government, not-for-profit organisations that provide welfare services to disadvantaged individuals. SCSOs hold considerable potential for providing smoking cessation support to disadvantaged smokers. This study aimed to establish the prevalence of smoking, interest in quitting and interest in receiving cessation support amongst clients accessing SCSOs.

**Methods:**

Clients seeking financial or material assistance from three SCSOs in NSW, Australia, between February and October 2010 were invited to complete a 60-item general health touch screen computer survey. This included questions about smoking status, past quit attempts and interest in receiving support to quit smoking from SCSO staff.

**Results:**

A total of 552 clients were approached to participate during the study period, of which 383 provided consent and completed the survey (69% consent rate). Daily smoking was reported by 53.5% of participants. Occasional smoking (non-daily smoking) was reported by a further 7.9% of participants. Most participants had tried to quit smoking in the past (77%) and had made an average of two quit attempts (SD = 3.2) lasting longer than 24 hours in the previous 12 months. More than half of all participants (52.8%) reported that they would like help from SCSO staff to quit smoking. For those interested in receiving help, the preferred types of help were access to free NRT (77%), cash rewards (52%) and non-cash rewards (47%) for quitting, and to receive support and encouragement from SCSO staff to quit (45%).

**Conclusions:**

Smoking rates among clients accessing SCSO are substantially higher than the general population rate of 15.1%. A substantial proportion of clients are interested in quitting and want support from the SCSO to do so.

## Background

In 2009, the National Preventative Taskforce recommended that daily smoking prevalence in Australia be reduced to less than 10% by 2020 [1]. In recognition of high smoking rates among disadvantaged groups [2,3], the taskforce acknowledged that "*a special focus on working with and supporting disadvantaged groups and communities*" would be needed to achieve this target [1]. There has also been increasing international recognition of the need for policies and strategies to increase access, affordability and use of smoking cessation services and treatments by disadvantaged smokers [2,4,5]. While the importance of a comprehensive population level approach to tobacco control cannot be overstated, in 2008 the US guidelines for tobacco dependence treatment called for research to explore the effectiveness of novel treatment delivery settings, including community-based settings, for reaching low socioeconomic status smokers and those with limited formal education [6]. One novel setting with considerable potential in Australia is social and community service organisations (SCSOs).

SCSOs are non-government, not-for-profit organisations that provide welfare services including financial and family counselling, temporary accommodation, food and material aid, and child and family support. They have existing contact with a large number of disadvantaged groups including the homeless, individuals with a mental illness, the unemployed and Aboriginal and Torres Strait Islanders [7], and are uniquely placed to provide smoking cessation support to disadvantaged smokers; they are able to address smoking in a holistic way alongside other issues faced by their clients, can provide personalised ongoing support, and have demonstrated growing interest in this opportunity via participation in programs such as the Cancer Council NSW's Tackling Tobacco initiative (see http://www.cancercouncil.com.au/editorial.asp?pageid=2210). Qualitative and quantitative work has established the acceptability of providing and receiving smoking cessation support in the SCSO setting [8,9]. A small pilot study has also shown that providing training to staff of SCSOs develops confidence, skills and knowledge in addressing tobacco issues [10], overcoming some of the barriers identified in providing support in this setting [8]. While SCSO appear to be a promising setting for targeting disadvantaged smokers, no data exists to describe the prevalence of smoking and interest in quitting among clients attending SCSOs in order to make judgements about the potential reach of this approach.

### Objective

To describe the smoking prevalence, interest in quitting and interest in receiving smoking cessation support among clients accessing SCSOs for welfare support.

## Method

### Design & Sample

A cross-sectional health survey was conducted between February and October 2010 in two SCSOs located in Sydney, and one SCSO located in a regional area of NSW, Australia. Participants were clients seeking financial or material assistance such as food vouchers, free grocery items, or assistance paying bills or purchasing medications from the SCSO. Clients who were aged over 18 years, able to speak and/or read English, and who were not judged to be distressed or ill by the caseworker recruiting participants were eligible to participate.

### Recruitment & Procedure

A top down approach to recruitment of services was used. The Chief Executive Officer (CEO) of a large SCSO operating in NSW, Australia, was initially approached for consent for the organisation to be involved in the research. The CEO nominated services to participate, who were then contacted for permission to be involved. Eligible service attendees were invited by their caseworker at the end of their appointment seeking financial or material assistance to complete a confidential and anonymous touch screen computer health survey. Gender and date of birth of non-consenting clients was collected to assess participation bias. Support to read and/or complete the touch screen computer survey of health status was provided by a research assistant when necessary. Ethics approval was provided by the University of Newcastle Human Research Ethics Committee.

### Measures

Participants completed a 60-item general health survey which included items on smoking, fruit and vegetable consumption, sun protection, physical activity, alcohol consumption and cancer screening. Only items related to smoking will be reported here. All questions were presented on a touch screen computer using Digivey survey software [11]. Questions related to:

1. *Socio-demographics: *gender, age, income, Aboriginal or Torres Strait islander status, employment and highest level of education.

2. *Smoking behaviours: *Smoking status was assessed by asking "Do you currently smoke tobacco products?" with response options i) 'Yes, daily', ii) 'Yes, at least once a week', iii) 'Yes, but less often than once per week' and iv) 'No, not at all'. Those reporting daily or occasional smoking were asked about the type of tobacco used and the average amount spent on tobacco each week ($AUD). Those reporting daily smoking were asked the age they first started smoking daily and, to enable the calculation of the heaviness of smoking index (HSI), were asked to report the number of cigarettes smoked each day, and time to first cigarette after waking [12]. Those who reported not smoking were asked if they had ever been a daily smoker (yes/no), and if so, how long ago they had quit.

3. *Smoking induced financial deprivation: *was assessed by asking participants "In the last six months, have you spent money on cigarettes that you knew would be better spent on household essentials like food?" (yes/no) [13].

4. *Quitting behaviours*: Current smokers were asked whether they had ever tried to quit smoking (yes/no), the number of quit attempts lasting at least 24 hours in the past 12 months, who had advised to them to quit smoking, what strategies they had used to try and quit in the past and their interest and intention to quit.

5. *Interest in receiving quit support from SCSOs: *Current smokers were asked whether they would be interested in receiving support to quit smoking from organisation staff (yes/no) and the type of support wanted (12 possible response options).

### Statistical Analysis

Frequencies were calculated and Chi-square tests used to examine differences between smokers and non-smokers using categorical data. HSI was calculated to give a score with a range of 0 (low dependence) to 6 (high dependence). Statistical analysis was conducted using STATA version 11.0 [14].

## Results

### Characteristics of the sample

A total of 552 clients were approached to participate during the study period, of which 383 completed the survey (69% consent rate). There were no differences in age between those who did (M = 43, SD = 12.6) and did not (M = 42.9, SD = 12.3) consent to participate, however male participants were more likely than female participants to agree to participate (76% vs. 67% respectively, χ^2 ^= 5.5, p = 0.02). Demographic details are reported in table 1. The majority of participants reported an income of less than AUD$300 per week, were unemployed and reported primary or secondary school as their highest level of education.

**Table 1:** Demographic characteristics of respondents (N = 383)

	Smokers (n = 235) % (95%)	Non-Smokers (n = 148) % (95%)	Total Sample (N = 383) % (95%)	χ^2^
**Gender**				
Male	60.4 (54.1-66.7)	46.6 (38.5-54.7)	55.1 (50.1-60.1)	χ^2 ^= 7, p < 0.01
Female	39.6 (33.3-45.9)	53.4 (45.3-61.5)	44.9 (39.9-49.9)	
**Age**				
< 29	13.2 (8.8-17.5)	12.8 (7.3-18.3)	13.0 (9.7-16.4)	χ^2 ^= 18.5, p < 0.01
30-39	28.9 (23.1-34.8)	21.6 (14.9-28.3)	26.1 (21.7-30.5)	
40-49	31.5 (25.5-37.5)	23.6 (16.8-30.5)	28.5 (23.9-33.0)	
50-59	20.4 (15.2-25.6)	23.6 (16.8-30.5)	21.7 (17.5-25.8)	
60-69	4.3 (1.7-6.8)	9.5 (4.7-14.2)	6.3 (3.8-8.7)	
70+	1.7 (0.04-3.4)	8.8 (4.2-13.4)	4.4 (2.4-6.5)	
**Aboriginal or Torres Strait Islander**				
Yes	12.3 (8.1-16.6)	8.8 (4.2-13.4)	11 (7.8-14.1)	χ^2 ^= 1.2, p = 0.28
No	87.7 (83.4-91.9)	91.2 (86.6-95.8)	89 (85.9-92.1)	
**Marital Status**				
Married	5.5 (2.6-8.5)	11.5 (6.3-16.7)	7.8 (5.1-10.5)	χ^2 ^= 16.4, p < 0.01
Defacto	8.5 (4.9-12.1)	5.4 (1.7-9.1)	7.3 (4.7-9.9)	
Separated/divorced	23 (17.6-28.4)	27.7 (20.4-34.9)	24.8 (20.5-29.1)	
Never married	59.6 (53.3-65.9)	45.3 (37.2-53.3)	54 (49-59)	
Widowed	3.4 (1.1-5.7)	10.1 (5.2-15.0)	6 (3.6-8.4)	
**Education**				
Primary school	3 (0.7-5.2)	2.7 (0.07-5.3)	2.9 (1.2-4.6)	χ^2 ^= 13.4, p < 0.01
High school 7-10	53.2 (46.8-59.6)	35.1 (27.4-42.9)	46.2 (41.2-51.2)	
High school 11-12	16.2 (11.4-20.9)	19.7 (13.2-26.1)	17.5 (13.7-21.3)	
TAFE	15.3 (10.7-19.9)	21.6 (14.9-28.3)	17.7 (13.9-21.6)	
University Degree	12.3 (8.1-16.6)	20.9 (14.3-27.5)	15.7 (12.0-19.3)	
**Income**				
< $200	18.3 (13.3-23.3)	12.8 (7.4-18.3)	16.2 (12.5-19.9)	χ^2 ^= 3.9, p = 0.42
$200-$300	36.2 (30.0-42.3)	38.5 (30.6-46.4)	37.1 (32.2-41.9)	
$300-$400	25.5 (19.9-31.1)	24.3 (17.4-31.3)	25.1 (20.7-29.4)	
$400-$500	9.4 (5.6-13.1)	8.1 (3.7-12.5)	8.9 (6.0-11.7)	
> $500	5.1 (2.3-7.9)	8.9 (4.2-13.4)	6.5 (4.0-9.0)	
Missing	5.5 (2.6-8.5)	7.4 (3.2-11.7)	6.2 (3.8-8.7)	
**Employment**				
Full time	1.3 (0.3-3.7)	0.7 (0.2-3.7)	1 (0.02-2.1)	χ^2 ^= 8.2, p = 0.32
Part time or casual	6.4 (3.2-9.5)	7.4 (3.2-11.7)	6.8 (4.3-9.3)	
Unemployed	48.5 (42.1-54.9)	49.3 (41.2-57.4)	48.8 (43.8-53.9)	
Student	4.2 (1.7-6.8)	6 (2.2-10)	5 (2.8-7.1)	
Retired	2.9 (0.8-5.2)	7.4 (3.2-11.7)	4.8 (2.6-6.8)	
Unable to work	12.8 (8.5-17.1)	12.2 (6.9-17.5)	12.5 (9.2-15.9)	
Home duties	11.1 (7.0-15.1)	10.1 (5.2-15.0)	10.7 (7.6-13.8)	
Other	12.8 (8.5-17.1)	6.9 (2.7-10.8)	10.4 (7.4-13.5)	

### Smoking behaviours

Smoking characteristics of the sample are reported in table 2. More than half of all participants (53.5%) reported daily smoking. A further 7.9% were occasional smokers. Of those who reported being an ex-smoker, the majority (57.4%) had quit smoking longer than 5 years ago. Males were more likely to smoke than females (67% v. 54%). Younger participants, those who were never married or single, and those with a high school year 7-10 education were also significantly more likely to smoke than their counterparts. Ex-smokers were more likely to be female (χ^2 ^= 4.7, p = 0.03). 78% of participants reported that they had been near others who were smoking in the past 24 hours and 61% of smokers reported that they had spent money on cigarettes they knew would be better spent on household essentials like food in the past six months.

**Table 2:** Smoking characteristics of the study sample (n = 235).

	% (95% CI)
**Smoking status**	
Daily	53.5 (48.5-58.5)
Weekly	4.2 (2.2-6.2)
Less than weekly	3.7 (1.8-5.5)
Never-smoker	22.4 (18.3-26.7)
Ex-smoker	16.2 (12.5-19.9)
**HSI**	
Low	36.5 (29.8-43.1)
Moderate	44.3 (37.4-51.2)
High	19.2 (13.7-24.7)
**Smoking induced financial deprivation**	
Yes	61.3 (55-67.6)
No	38.7 (32.4-45)

	**Mean (SD)**

**Age started smoking**	
Males	15.7 (4.4)
Females	17.7 (7)
**Number of cigarettes smoked daily**	16.8 (10.6)
**Amount spent on cigarettes weekly ($AUD)**	42.9 (31.1)

### Quitting

Quitting behaviours are reported in table 3. Overall, 77% of participants had tried to quit smoking in the past. Participants had made an average of 2.1 quit attempts lasting longer than 24 hours in the previous 12 months (SD = 3.2; range 0-20). The majority had attempted to quit cold turkey (74%). A minority had used NRT (32.9%), or called Quitline (7.7%). More than half of participants were 'very' or 'quite' interested in quitting smoking (56.6%), however relatively few intended to quit in the next 30 days (16.2%).

**Table 3:** Quitting behaviours and intentions among sample of daily and occasional smokers (n = 235 unless otherwise noted)

	% (95% CI)
**Interest in quitting**	
Very interested	36.2 (30.0-42.4)
Quite interested	20.4 (15.2-25.6)
A little bit interested	19.6 (14.5-24.7)
Not at all interested	23.8 (18.3-29.3)
**Intention to quit**	
Next 30 days	16.2 (11.4-20.9)
Next 6 months	25.9 (20.3-31.6)
Quit, but not in next 6 months	17.9 (12.9-22.8)
Never quit	6.8 (3.7-10.0)
Don't know	33.2 (27.1-39.3)
Who has advised to quit *	
Doctor	38.7 (32.4-45.0)
Family member	38.7 (32.4-45.0)
No one	37.0 (30.8-43.2)
Friend	26.4 (20.7-32.1)
Other	11.1 (7.0-15.1)
Nurse	6.0 (2.9-9.0)
Caseworker	6.0 (2.9-9.0)
Teacher	2.1 (0.2-4.0)
Boss	3.4 (1.2-5.7)
Quit strategies used in the past*^	
Cold turkey	74 (67.6-80.5)
Used NRT	39.2 (32.0-46.4)
Other	19.3 (13.5-25.1)
Received support from family/friends	8.3 (4.2-12.3)
Called Quitline	7.7 (3.8-11.7)
Acupuncture or hypnosis	5 (1.7-8.2)
Individual counselling	2.8 (0.3-5.2)
Group quit program	0.5 (0.04-1.6)

*Participants could select more than one response. Percentages do not add to 100%.

^Answered only by participants who reported making a quit attempt, n = 181.

### Interest in receiving quit support from SCSOs

Just over half of all participants (52.8%) reported that they would like help from community service staff to quit smoking. Types of help wanted are shown in table 4. For those wanting support, the most desired types were access to free NRT (77.4%), cash rewards (52.4%) and non-cash rewards (46.8%) for quitting, and to receive support and encouragement from SCSO staff to quit (45.2%). The least desired types of support were to be put in touch with the telephone Quitline (11.3%) and to receive quit help via SMS messages (12.9%).

**Table 4:** Types of quit support most desired by clients who wanted support from SCSO staff to quit (n = 124)

	% (95% CI)
Be given free nicotine patches or gum	77.4 (70.0-84.9)
Be given cash rewards for quitting	52.4 (43.5-61.3)
Be given non-cash rewards for quitting	46.8 (37.9-55.7)
Get support and encouragement from staff to quit	45.2 (36.3-54.1)
Alternative therapy like acupuncture or hypnosis	38.7 (30.0-47.4)
Receive advice or counselling	31.5 (23.2-39.7)
Be asked by staff if I would like help to quit	31.5 (23.2-39.7)
Be given pamphlets about quitting	23.4 (15.8-30.9)
Computer or internet based quit program	15.3 (8.9-21.8)
Video or DVD about quitting	14.5 (8.2-20.8)
Quit help via SMS messages	12.9 (6.9-18.9)
Be put in touch with Quitline	11.3 (5.6-16.9)

## Discussion

The rate of current daily smoking at 53.5% was more than three times higher than the Australian population rate of 15.1% [15], and comparable to that documented in other severely disadvantaged groups such as those attending a psychiatric rehabilitation support service [16]. Daily consumption of cigarettes at 16.7 per day was slightly higher than the general population consumption of 13.9 cigarettes per day [17]. A considerably smaller proportion of participants were never smokers compared to the general population [17]. These data confirm that SCSO clients have rates of smoking and nicotine dependence similar to that of the most disadvantaged groups in Australia.

A high proportion of smokers had attempted to quit in the past year, adding further support to evidence that disadvantaged smokers have a desire to quit smoking that is comparable to the general population [18]. However, a relatively small proportion of participants reported using strategies known to increase quit success, including using nicotine replacement therapy and behavioural support. Few participants had contacted the telephone Quitline, and few showed interest in receiving this type of support. Alarmingly, over a third of respondents wanted help from the SCSO to access acupuncture and hypnosis, despite there being no evidence of the effectiveness of these types of support [19]. While the cost of nicotine replacement therapy is sometimes reported as a barrier to use amongst disadvantaged smokers and could explain this finding [20], further exploration of the reasons why disadvantaged smokers do not use other available services such as the telephone Quitline is needed. Such work would help inform the developed of strategies to increase engagement of disadvantaged smokers with evidence-based cessation interventions that will increase the likelihood of quit success.

More than half of smokers wanted support from the SCSO to quit, which highlights the potential of SCSOs to reach disadvantaged smokers. The Australian Council of Social Services reports that member SCSOs provided services to disadvantaged clients on more than 4.3 million occasions in 2009 [7]. Assuming a smoking rate of 62% and that 53% of clients would accept support, SCSOs could provide support to smokers on nearly 1.5 million occasions each year. SCSO client populations contain an over-representation of single parents, Aboriginal and Torres Strait Islanders, and individuals receiving social welfare payments [7], providing a unique way to access the most disadvantaged smokers in the community. It is unclear however whether utilization of support provided by SCSOs would be more or less than the 53% suggested by our data. Large randomised controlled trials are needed to examine the uptake of support by clients in this setting, and the effectiveness of this approach in increasing smoking cessation. A trial examining the efficacy of a client-centred, caseworker-delivered cessation support intervention is currently underway [21].

## Conclusions

Smoking rates among clients accessing SCSOs are markedly higher than the general population. Given that a high proportion of smokers are interested in receiving quit support from SCSOs, the effectiveness of integrating the delivery of evidence-based support into care provided by SCSOs should be further explored.

## Competing interests

The authors declare that they have no competing interests.

## Authors' contributions

JB, BB, and CP conceived of the study and were involved in its design and co-ordination. JB and BB supervised data collection. Statistical analysis was carried out by JB and CL. JB led manuscript preparation. All authors were involved in data interpretation and revised the manuscript critically for intellectual content. All authors approve of the final version of the manuscript.

## Pre-publication history

The pre-publication history for this paper can be accessed here:

http://www.biomedcentral.com/1471-2458/11/827/prepub
